# Coherence Analysis of Cardiovascular Signals for Detecting Early Diabetic Cardiac Autonomic Neuropathy: Insights into Glycemic Control

**DOI:** 10.3390/diagnostics15121474

**Published:** 2025-06-10

**Authors:** Yu-Chen Chen, Wei-Min Liu, Hsin-Ru Liu, Huai-Ren Chang, Po-Wei Chen, An-Bang Liu

**Affiliations:** 1 Department of Neurosurgery, Hualien Tzu Chi Hospital, Buddhist Tzu Chi Medical Foundation, Hualien 970473, Taiwan; spring810569@gmail.com; 2 Department of Medical Informatics, Tzu Chi University, Hualien 970374, Taiwan; 3 Department of Biomedical Sciences and Engineering, Tzu Chi University, Hualien 970374, Taiwan; 4 Department of Computer Science and Information Engineering, National Chung Cheng University, Chiayi 621301, Taiwan; wmliu@cs.ccu.edu.tw; 5 Department of Medical Research, Hualien Tzu Chi Hospital, Hualien 970473, Taiwan; stylecindy@gmail.com; 6 Division of Cardiology, Department of Internal Medicine, Hualien Tzu Chi Hospital, Buddhist Tzu Chi Medical Foundation, Hualien 970473, Taiwan; huairenchang@mail.tcu.edu.tw; 7 Department of Medicine, School of Medicine, Tzu Chi University, Hualien 970473, Taiwan; 8 Department of Physical Medicine and Rehabilitation, Hualien Tzu Chi Hospital, Buddhist Tzu Chi Medical Foundation, Hualien 970473, Taiwan; drpwchen@gmail.com; 9 Department of Neurology, Hualien Tzu Chi Hospital, Buddhist Tzu Chi Medical Foundation, Hualien 970473, Taiwan

**Keywords:** diabetic cardiac autonomic neuropathy, frequency-domain coherence analysis, heart rate variability, baroreflex sensitivity, glycemic control

## Abstract

**Background:** Cardiac autonomic neuropathy (CAN) is a common yet frequently underdiagnosed complication of diabetes. While our previous study demonstrated the utility of multiscale cross-approximate entropy (MS-CXApEn) in detecting early CAN, the present study further investigates the use of frequency-domain coherence analysis between systolic blood pressure (SBP) and R-R intervals (RRI) and evaluates the effects of insulin treatment on autonomic function in diabetic rats. **Methods:** At the onset of diabetes induced by streptozotocin (STZ), rats were assessed for cardiovascular autonomic function both before and after insulin treatment. Spectral and coherence analyses were performed to evaluate baroreflex function and autonomic regulation. Parameters assessed included low-frequency power (LFP) and high-frequency power (HFP) of heart rate variability, coherence between SBP and RRI at low and high-frequency bands (LF_Coh_ and HF_Coh_), spontaneous and phenylephrine-induced baroreflex sensitivity (BRS_spn_ and BRS_phe_), HRV components derived from fast Fourier transform, and MS-CXApEn at multiple scales. **Results:** Compared to normal controls (LF_Coh_: 0.14 ± 0.07, HF_Coh_: 0.19 ± 0.06), early diabetic rats exhibited a significant reduction in both LF_Coh_ (0.08 ± 0.04, *p* < 0.05) and HF_Coh_ (0.16 ± 0.10, *p* > 0.05), indicating impaired autonomic modulation. Insulin treatment led to a recovery of LF_Coh_ (0.11 ± 0.04) and HF_Coh_ (0.24 ± 0.12), though differences remained statistically insignificant (*p* > 0.05 vs. normal). Additionally, low-frequency LFP increased at the onset of diabetes and decreased after insulin therapy in most rats significantly, while MS-CXApEn at all scale levels increased in the early diabetic rats, and MS-CXApEn_large_ declined following hyperglycemia correction. The BRS_spn_ and BRS_phe_ showed no consistent trend. **Conclusions:** Coherence analysis provides valuable insights into autonomic dysfunction in early diabetes. The significant reduction in LF_Coh_ in early diabetes supports its role as a potential marker for CAN. Although insulin treatment partially improved coherence, the lack of full recovery suggests persistent autonomic impairment despite glycemic correction. These findings underscore the importance of early detection and long-term management strategies for diabetic CAN.

## 1. Introduction

Diabetes mellitus is one of the most prevalent chronic diseases worldwide and a leading cause of mortality [1,2]. Among its numerous complications, cardiac autonomic neuropathy (CAN) is recognized as one of the most severe [3]. The occurrence of diabetic CAN varies depending on the demographic factors, methodology and diagnostic criteria [4]. CAN not only disrupts the autonomic innervation of the heart but also impairs blood vessels and the regulatory circuits between the heart and vasculature. This disruption compromises cardiovascular homeostasis, leading to complications such as cardiac arrhythmias, postural hypotension, and hypertension [5,6,7]. The prevalence of CAN in diabetes varies from 7.7% to 99%, depending on the diagnostic criteria and assessment methods [3]. Several methods have been developed for detecting and evaluating CAN [8]. Heart rate variability (HRV) and baroreflex sensitivity (BRS) are two commonly used techniques for assessing diabetic CAN.

Heart rate variability refers to the physiological variation in the time intervals between consecutive heartbeats, known as R-R intervals (RRIs). It serves as a non-invasive biomarker of autonomic nervous system function, reflecting the dynamic interplay between sympathetic and parasympathetic modulation of cardiac activity [9,10]. HRV can be assessed using various analytical approaches, primarily categorized into time-domain, frequency-domain, and nonlinear methods [11]. Frequency-domain analysis, commonly performed using fast Fourier transform (FFT), is widely applied in both research and clinical settings. This method decomposes HRV signals into spectral components associated with autonomic regulation. The low-frequency (LF) component (0.04–0.15 Hz) reflects both sympathetic and parasympathetic activity, whereas the high-frequency (HF) component (0.15–0.4 Hz) is predominantly linked to vagal modulation of the heart. The LF/HF ratio has been frequently used as an index of sympathovagal balance, although its interpretation remains a topic of debate [12]. Beyond traditional spectral analysis, nonlinear methods such as Poincaré plot analysis, entropy measures, and detrended fluctuation analysis have been introduced to capture the complexity and fractal-like properties of HRV. These approaches provide deeper insights into autonomic regulation and physiological adaptability. Importantly, nonlinear metrics have demonstrated potential in detecting early autonomic dysfunction in conditions such as diabetes, cardiovascular disease, and neurodegenerative disorders, offering valuable prognostic and diagnostic information.

The baroreflex maintains cardiovascular homeostasis by adjusting heart rate (HR) in response to systolic blood pressure (SBP) fluctuations. Baroreceptors in the aortic arch and carotid sinus detect SBP changes, transmitting signals to the brainstem. Increased SBP enhances parasympathetic activity and suppresses sympathetic output, prolonging pulse-to-pulse intervals (PPI) and reducing heart rate (HR), while decreased SBP elicits the opposite response. These dynamic SBP–PPI interactions reflect baroreceptor–autonomic coupling, crucial for BP stabilization [9]. The baroreflex activity, referred to as baroreflex sensitivity (BRS), can be assessed using time-domain and frequency-domain methods.

Time-domain techniques, such as the sequence method or pharmacological intervention (Oxford method) offer real-time and computationally efficient evaluations of BRS. These methods typically analyze beat-to-beat relationships between SBP and inter-beat intervals, making them suitable for continuous clinical monitoring due to their simplicity and responsiveness. These provide significantly faster computation, making it ideal for long-term clinical applications [13]. In contrast, frequency-domain methods, including spectral decomposition and transfer function analysis, assess BRS within specific frequency bands—most notably the low-frequency range, which corresponds to the primary operational bandwidth of the baroreflex. These methods provide deeper insights into the dynamics of autonomic regulation and are particularly adept at detecting subtle physiological alterations, such as diminished baroreflex gain in pathological states like acute myocardial infarction [14].

Frequency coherence is a fundamental signal processing technique used across various scientific disciplines to assess the phase relationship between two signals across different frequency ranges. In oceanography, coherence analysis plays a crucial role in studying interactions between ocean currents, sea surface temperature variations, and climate patterns such as the El Niño-Southern Oscillation (ENSO) [15]. It helps researchers understand how environmental factors influence ocean circulation and energy transfer. Similarly, in meteorology, coherence analysis is employed to examine the coupling between atmospheric pressure fluctuations and weather patterns, aiding in climate modeling and long-term forecasting [12]. Beyond environmental sciences, coherence analysis has found widespread applications in biomedical research. In neuroscience, it is used to analyze electroencephalogram (EEG) signals to assess functional connectivity between different brain regions, offering valuable insights into neurological disorders [16]. In cardiovascular physiology, coherence analysis provides a complementary approach for evaluating the frequency-dependent coupling between SBP and RRIs, offering an alternative method for assessing baroreflex function. This study aims to investigate alterations in LF and HF coherence in diabetic rats, explore the potential effects of glycemic correction on cardiovascular autonomic function, and compare coherence analysis with established BRS measures, including phenylephrine-induced BRS (BRS_phe_), BRS_spn_, and the nonlinear method of multiscale cross-approximate entropy (MS-CXApEn) [17]. By integrating coherence analysis with conventional BRS assessments, we seek to determine their respective advantages and applicability in detecting early autonomic dysfunction in diabetes.

## 2. Materials and Methods

### 2.1. Animals

Adult male Wistar Kyoto (WKY) rats, aged approximately 8 weeks old, were obtained from the National Laboratory Animal Breeding and Research Center in Taipei, Taiwan. The animals were housed at the Tzu Chi University Animal Center in a temperature- and humidity-controlled environment (22–24 °C) under a 12-h light-dark cycle, with free access to standard rat chow and water ad libitum. Stress-related factors may secondarily affect baroreflex sensitivity and cardiovascular autonomic control. To minimize the potential confounding effects of housing density on autonomic function, animals were housed in pairs (two per cage) to maintain social contact while minimizing stress-induced variability during the acclimatization. During the induction period and physiological signal acquisition, each rat was individually housed in a separate cage to avoid stress-induced alterations in cardiovascular autonomic parameters [18,19]. This housing strategy was adopted in consideration of prior studies indicating that social isolation or overcrowding can influence the hypothalamic–pituitary–adrenal (HPA) axis, sympathetic activity, and related autonomic outcomes in rodent models [20]. All experimental procedures adhered to ethical guidelines and were approved by the Institutional Animal Care and Use Committee of Tzu Chi General Hospital (IACUC: 102-29). The study followed the Guide for the Care and Use of Laboratory Animals (NIH publication no. 85-23, revised 1996), published by the National Academy Press, Washington, DC, USA.

### 2.2. Induction of Diabetes

Diabetes was induced in eight WKY rats through an intraperitoneal injection of nicotinamide (180 mg/kg, dissolved in 0.9% saline; Sigma, St. Louis, MO, USA), followed 30 min later by an injection of streptozotocin (STZ, 50 mg/kg; Sigma). Blood glucose levels were monitored daily using a Contour Plus^®^ glucose meter (Bayer HealthCare, Leverkusen, North Rhine-Westphalia, Germany). Diabetes was defined as a random blood glucose level exceeding 250 mg/dL [21], which was typically observed 5–7 days after the induction procedure.

### 2.3. Surgical Procedures and Acquisition of Physiological Signals

The surgical and physiological signal acquisition methods followed previously published protocols [22]. In brief, animals were anesthetized with intraperitoneal urethane (1500 mg/kg) and positioned supine for the procedures. A PE-50 polyethylene catheter was inserted into the right femoral artery and connected to a pressure transducer to monitor arterial blood pressure. A second catheter was placed in the left femoral vein for pharmacological interventions. Lead II ECG signals were recorded using disposable twisted subdermal needle electrodes positioned on the bilateral forelimbs and tail. Both blood pressure and ECG signals were captured synchronously with the SINGA^®^ Xction View II system (Diagnostic and Research Instruments Co., Ltd., Chiayi, Taiwan) at a 1000 Hz sampling rate. Signals were continuously monitored to ensure baseline stability and were stored for subsequent analysis. All animals were kept on a heated surgical platform to maintain body temperature during the procedure. No additional fluid replacement or analgesia was provided due to the brief duration and minimal invasiveness of the procedures.

### 2.4. Evaluation of Baroreflex in the Time Domain

**Figure 1:**
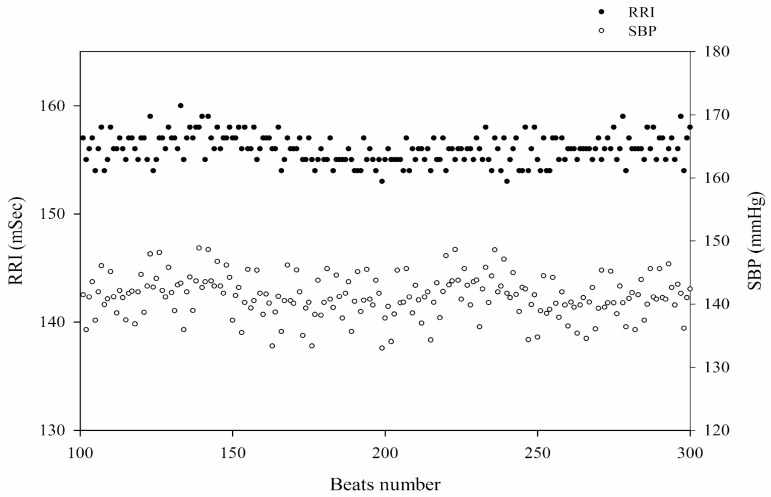
Beat-to-beat fluctuations of systolic blood pressure (SBP) and R-R interval (RRI) represented by discrete data points in a normal WKY rat. The use of discrete points emphasizes the variability in SBP and RRI, facilitating a more precise assessment of beat-to-beat interactions.

After equilibrium, spontaneous oscillations of SBP with corresponding RRI were observed during a stable long-term recording (Figure 1). To assess (BRS_spn_), a five-minute segment of stable SBP and the corresponding RRIs was selected. Based on the baroreflex mechanism, each relevant RRI was defined as the consecutive RRI following the synchronously recorded SBP. The detailed methodology has been described previously [22]. Briefly, BRS_spn_ was quantified using the sequence method as the mean correlation between SBP and its corresponding RRI. Sequences with a slope > 0.85 were included in the final analysis, and BRS_spn_ was determined as the mean slope of all valid sequences. The pharmacological intervention was based on the idea of the Oxford method [10]. Following a 30-min stabilization period for physiological signal acquisition, baroreflex sensitivity was assessed through pharmacological intervention. A bolus injection of 100 µL phenylephrine (8.0 µg/mL, Sigma-Aldrich, St. Louis, MO, USA) was administered intravenously via the left femoral vein to induce a 20–40 mmHg increase in SBP above baseline. Baroreflex sensitivity (BRS_phe_) was determined by analyzing the linear regression between SBP changes (ΔSBP, mmHg) and the corresponding prolongation of R-R intervals (ΔRRI, msec). Only sequences with at least four consecutive increases in SBP and a correlation coefficient exceeding 0.8 were considered valid for analysis [22].

### 2.5. Evaluation of Cardiac Autonomic Function in the Frequency Domain

These spectral features of RRI were derived using fast Fourier transform (FFT) analysis as our previous publication [17]. The frequency ranges are associated with heart rate dynamics and beat-to-beat variability. Unlike human HRV, where standard frequency bands are well-defined, HRV analysis in rats follows distinct spectral ranges. Specifically, the LF component is defined within 0.20–0.75 Hz, while the HF component spans 0.75–5.0 Hz. These frequency bands have been widely adopted for evaluating autonomic regulation in rodent models [23].

### 2.6. Evaluation of Baroreflex Sensitivity Using Frequency Coherence Between SBP and RRI

The frequency domain analysis of SBP and RRI was conducted using custom-written MATLAB scripts (MATLAB 2019, MathWorks, Inc., Natick, MA, USA). To ensure temporal alignment, the signals were resampled at a uniform frequency of 10 Hz using spline interpolation, and linear trends were removed to mitigate artificial spectral leakage. All recorded signals exceeded 5 min in duration. The power spectral density (PSD) of RRI and SBP was estimated using the Welch method with a Hanning window and 50% overlap, yielding a spectral resolution of 0.005 Hz.

The relationship between RRI and SBP in the frequency domain was further assessed through coherence analysis. Coherence is a function of the PSD of *x* and *y* (*P_xx_* and *P_yy_*, respectively) and the cross PSD of *x* and *y* (*P_xy_*):

$$C_{xy}\left(f\right)=\;\frac{{|P}_{xy}\left(f\right){|}^{2}}{P_{xx}(f)P_{yy}(f)}.$$

The coherence function used the Welch method with a Hanning window (10 s sample numbers) and 50% overlap between windows that produced a 0.01 Hz spectral resolution. The significant coherence level in analyses was calculated as

*limit =* 1 *− (*1 *− α)*^1/*(N* − 1*)*^.

where *α* = 0.95, *n* denotes the number of independent frequencies under investigation and *N* is the number of 100 consecutive windows used for the coherence calculation. This allows for a confidence value of *p* < 0.05 when rejecting the null hypothesis of non-significant coherence [24]. The significant coherence within the frequency range of 0.08–1.5 Hz was averaged to represent low-frequency coherence (LF_Coh_), while coherence within the 1.51–5 Hz range was considered high-frequency coherence (HF_Coh_).

### 2.7. Study Design and Protocols

This study included eight WKY rats with early-stage STZ-induced diabetes and eight age-matched normoglycemic controls. Blood sugar levels were checked using blood sticks, Contour Plus^®^ after intravenous and intra-arterial cannulation. Then, blood pressure and ECG signals were recorded for 30 min after equilibration. A five-minute period of stable signals was used to assess BRS, HRV, and coherence. Pharmacological intervention of baroreflex activity was performed after the acquisition of baseline physiological signals. Early diabetes was defined as the first day on which hyperglycemia was confirmed, indicated by a blood glucose level exceeding 250 mg/dL, typically occurring 5–7 days after STZ injection. The diabetic rats received subcutaneous insulin (100 IU/mL) according to their glycemic status: 1 unit was administered if blood glucose exceeded 400 mg/dL, and 2 units were given if levels exceeded 600 mg/dL. Blood glucose was rechecked one hour after insulin administration. To evaluate changes in cardiac autonomic function under euglycemic conditions, a second recording of blood pressure and ECG signals was performed when blood glucose levels had decreased to below 200 mg/dL.

### 2.8. Statistical Analysis

All data are expressed as mean ± standard deviation (SD). Comparisons of body weight, blood pressure, heart rate, and parameters of cardiovascular autonomic function including LFP, HFP and LHR, BRS_spn_, BRS_phe_ and MS-CXApEn across various scales (small, medium, and large) were conducted using the Mann–Whitney U-test or one-way ANOVA with Fisher’s least significant difference (LSD) post-hoc test. After normalization of the raw data, Bland–Altman analysis was used to evaluate the agreement between BRS assessed by various methods and BRS_phe_ (the Oxford method) across two separate time periods. Good agreement was defined as the majority of differences falling within ±1.96 standard deviations (SD) of the mean difference. Statistical analyses were performed using STATA software (version 15.0, StataCorp LLC, College Station, TX, USA), and significance was set at *p* < 0.05.

## 3. Results

### 3.1. Physiological Parameters of Normal Rats, and Diabetic Rats in Hyperglycemic and Euglycemic Status

Table 1 provides a comparison of key physiological parameters among normal control rats and diabetic rats in both hyperglycemic and insulin-treated euglycemic states. Diabetic rats exhibited a significant reduction in body weight compared to their control counterparts at the onset of diabetes (303.75 ± 24.83 g vs. 265.25 ± 35.90 g). These diabetic rats also displayed markedly elevated blood glucose levels and altered cardiovascular parameters, including higher SBP and diastolic blood pressure (DBP) and an increased HR.

### 3.2. Agreements Physiological Parameters of Normal Rats, and Diabetic Rats in Hyperglycemic and Euglycemic Status

**Figure 2:**
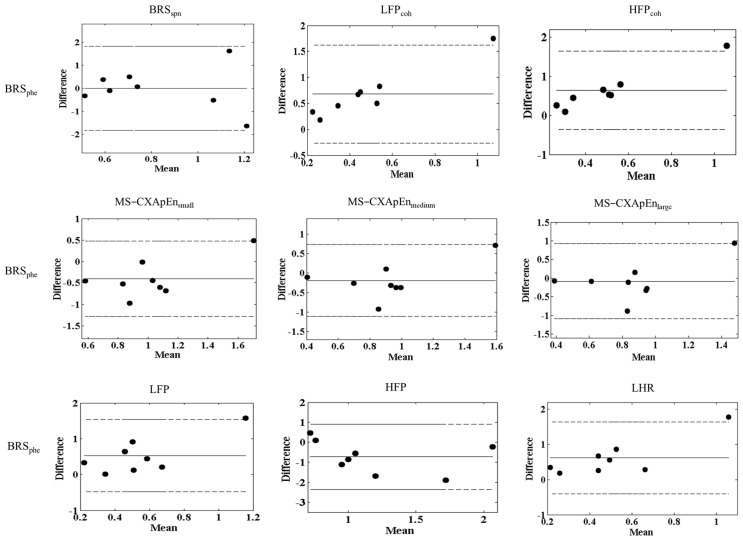
The Bland-Altman plots illustrate the agreement between baroreflex sensitivity assessed by using phenylephrine (BRS_phe_) and various cardiovascular autonomic parameters, including spontaneous baroreflex sensitivity (BRS_spn_), low and high-frequency coherence between systolic blood pressure (SBP) and R-R intervals (LFP_Coh_ and HFP_Coh_), multiscale cross-approximate entropy at small (MS-CXApEn_small_), medium (MS-CXApEn_medium_), and large scales (MS-CXApEn_large_), as well as heart rate variability components estimated by fast Fourier transform, including low-frequency power (LFP), high-frequency power (HFP), and their ratio (LHR). The mean difference (solid line) and limits of agreement (±1.96 standard deviations, dashed lines) are shown for each comparison. The closer the data points are to the mean difference line within the limits of agreement, the better the agreement between BRS_phe_ and the corresponding parameter.

Figure 2 presents Bland–Altman plots of BRS assessed by various methods against the gold standard Oxford method (BRS_phe_). The compared methods include BRS_spn_, LFP_Coh_, HFP_Coh_, MS-CXApEn at small, medium, and large scales, and spectral power indices of HRV (LFP, HFP, and LHR). Across the comparisons, the majority of data points fall within the mean difference ± 1.96 standard deviations, indicating overall good agreement between BRS_phe_ and the alternative methods. BRS_spn_ and HFP show the closest agreement with BRS_phe_, with a small mean difference and narrow limits of agreement. Similarly, MS-CXApEn-based assessments demonstrate strong agreement with BRS_phe_, particularly at medium and large scales, reinforcing their potential as alternative nonlinear markers of baroreflex function. Coherence-based BRS measures (LFP_Coh_, HFP_Coh_) also exhibit reasonable agreement with BRS_phe_, though with slightly wider limits of agreement. The spectral power indices (LFP, HFP, and LHR) show greater variability when compared with BRS_phe_. Notably, one outlier beyond the 1.96 SD range is observed in the HFP_Coh_ and MS-CXApEn_small_ comparisons, suggesting potential variability in specific cases.

### 3.3. Evaluations of Cardiovascular Autonomic Function in Early Diabetes

Table 2 compares cardiovascular autonomic functions between control rats and diabetic rats at the onset. Diabetic rats showed significant elevations in LFP (0.84 ± 0.64 vs. 0.29 ± 0.19) and the ratio of LFP to HFP (0.35 ± 0.10 vs. 0.20 ± 0.15) compared with normal controls. Moreover, diabetic rats exhibited significant elevations in MS-CXApEn_at all scales, suggesting increased complexity of the coupling between SBP and RRIs. Coherence at the low-frequency band was decreased in the diabetic rats. The other parameters, including BRS_spn_, BRS_phe_, and HFP, did not demonstrate statistically significant differences between groups.

However, BRS_phe_ and BRS_spn_, and HFP did not demonstrate statistically significant differences.

### 3.4. Changes of Cardiovascular Parameters Before and After Correction of Hyperglycemia in Eight Diabetic Rats

**Figure 3:**
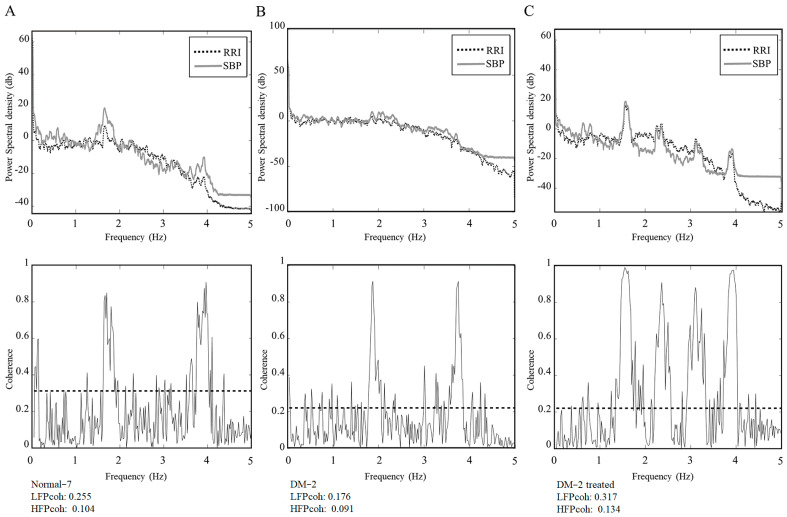
Power spectral density (PSD) and magnitude squared coherence analysis of systolic blood pressure (SBP) and R-R intervals (RRI) across three experimental conditions: (**A**) normal control (Normal-7), (**B**) diabetic rats with hyperglycemia (DM-2), and (**C**) diabetic rats receiving insulin therapy to restore euglycemia (DM-2 treated). The top row presents the PSD of SBP (solid gray line) and RRI (dashed black line), illustrating distinct spectral characteristics among the groups. The bottom row displays the magnitude squared coherence between SBP and RRI, with the dashed horizontal line indicating the statistical significance threshold (*p* < 0.05).

**Figure 4:**
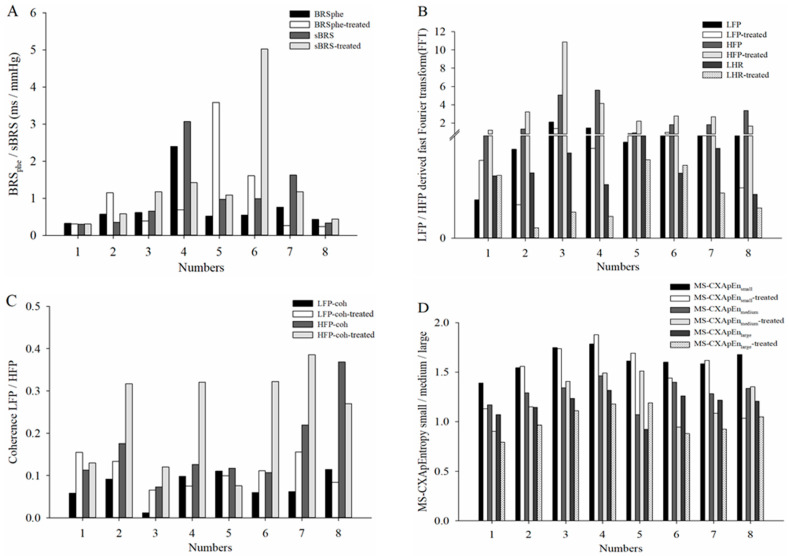
Changes in cardiovascular autonomic function parameters before and after insulin therapy in eight diabetic rats. (**A**) Baroreflex sensitivity (BRS) was assessed using phenylephrine-induced (BRS_phe_) and spontaneous baroreflex sensitivity (BRS_spn_). (**B**) Heart rate variability (HRV) components were estimated by fast Fourier transform, including low-frequency power (LFP), high-frequency power (HFP), and their ratio (LHR). (**C**) Low-frequency coherence (LF_Coh_) and high-frequency coherence (HF_Coh_) between systolic blood pressure (SBP) and R-R intervals (RRI). (**D**) Multiscale cross-approximate entropy (MS-CXApEn) at small, medium, and large scales.

Table 3 compares cardiovascular autonomic function parameters in diabetic rats under hyperglycemic and euglycemic conditions. Following insulin treatment to correct hyperglycemia, only LHR showed a significant decrease. Other parameters, including LFP, MS-CXApEn at different scales, LF_Coh_, and HF_Coh_, trended toward values observed in normal controls but did not reach statistical significance. Figure 3 illustrates the power spectral density (PSD) of SBP and RRI, along with the magnitude squared coherence between these signals, in these three exampled assessments: normal control (Normal-3), diabetic rats with hyperglycemia (DM-2), and diabetic rats treated with insulin to achieve euglycemia (DM-2 treated). In the PSD plots (top row), the spectral distribution of SBP (solid gray line) and RRI (dashed black line) exhibits distinct frequency characteristics across the groups. The normal control group demonstrated a well-defined spectral distribution, while the diabetic group with hyperglycemia (DM-2) showed altered spectral power, particularly in the LF and HF bands. Following insulin treatment (DM-2 treated), spectral patterns showed a partial trend toward normalization but did not fully match the control group. In the coherence plots (bottom row), the magnitude squared coherence between SBP and RRI is presented, with the dashed horizontal line representing the significance threshold (*p* < 0.05). The normal control group exhibited strong coherence in both LF and HF bands, suggesting intact baroreflex function. In contrast, the diabetic group with hyperglycemia (DM-2) displayed a reduction in coherence, particularly in the HF band, indicating impaired cardiac autonomic regulation. Insulin treatment led to a partial restoration of coherence in both frequency bands. Table 3 compares cardiovascular autonomic function parameters in diabetic rats before and after insulin treatment. After correcting hyperglycemia, only LHR decreased significantly. Other parameters, including LFP, MS-CXApEn at different scales, LF_Coh_, and HF_Coh_, showed trends toward normalization but without statistical significance. A rat-by-rat analysis was conducted to examine individual changes. Figure 4 illustrates the changes in cardiovascular autonomic function parameters before and after insulin therapy in eight diabetic rats. It reveals that LFP showed a reduction after insulin treatment in most rats. Both LF_Coh_ and HF_Coh_ exhibited an overall increasing trend following insulin therapy, though with variability among individuals. MS-CXApEn_large_ decreased in most diabetic rats after correcting hyperglycemia. The remaining parameters did not show a consistent pattern of change.

## 4. Discussion

Table 1 presents the physiological parameters of the study groups, including blood glucose levels, heart rate, SBP, DBP, and body weight. The early diabetic rats exhibited significantly elevated blood glucose levels and body weight loss compared to the normal control group. These changes are likely due to metabolic disturbances associated with diabetes, as commonly observed in clinical practice [25]. Furthermore, hyperglycemic diabetic rats displayed significant reductions in cardiovascular parameters, including SBP, DBP, and HR. These results align with previous observations indicating suppressed cardiovascular autonomic function during the early stage of streptozotocin-induced diabetes in rats, characterized by simultaneous decreases in blood pressure and HR. These physiological alterations imply autonomic dysregulation during early diabetic CAN. Consistent with earlier research, our data also suggest that hyperglycemia-induced autonomic dysfunction can initially manifest as paradoxical parasympathetic hyperactivity, resulting in bradycardia and reduced blood pressure prior to the subsequent sympathetic predominance observed in advanced disease stages [26,27]. Following the correction of hyperglycemia through insulin treatment, the euglycemic diabetic rats exhibited a trend toward normalization of cardiovascular parameters, including SBP, DBP, and HR. Although these values approached those of the normal controls, the differences were not statistically significant. This partial recovery highlights the potential reversibility of hyperglycemia-induced cardiovascular impairments with adequate glycemic control.

The findings of the Bland–Altman analyses [28] illustrate good general agreement between the Oxford pharmacological method (BRS_phe_) and other non-invasive autonomic function parameters evaluated in this study, validating the reliability and clinical applicability of these alternative assessments for BRS (Figure 2). Such agreements support the clinical relevance of spontaneous BRS methods, frequency coherence, and multiscale entropy analyses, which offer non-invasive alternatives for autonomic function assessment. However, the notable consistent outlier (Normal-8) emphasizes that individual variability or methodological inconsistencies can significantly impact the reproducibility of these measurements. Possible reasons for this outlier include individual variations in baroreflex sensitivity, physiological differences, or transient measurement errors. This observation underscores the need for careful data interpretation, particularly in small-sample experimental studies, where outliers can disproportionately influence statistical agreement. Repeated measurements or larger sample sizes might be necessary to mitigate such individual variability in future studies [29].

Heart rate variability and baroreflex sensitivity are widely employed to evaluate CAN in diabetic patients. HRV quantifies autonomic modulation of the heart by analyzing beat-to-beat variations in heart rate, providing insight into the overall autonomic balance between sympathetic and parasympathetic systems. Meanwhile, BRS specifically measures the responsiveness of the baroreflex, assessing reflexive heart rate adjustments in response to changes in arterial pressure. Utilization of HRV offers a comprehensive evaluation of cardiac autonomic function and enhances the sensitivity for detecting early-stage autonomic dysfunction in diabetes [30]. Table 2 summarizes the cardiovascular autonomic function parameters in control and diabetic rats under hyperglycemic conditions. Compared with normal controls, diabetic rats showed significantly decreased LF_Coh_ accompanied by increased LFP, HFP and LHR, and MS-CXApEn at all scales. These findings indicate disrupted cardiovascular autonomic regulation in diabetic rats during hyperglycemia. Previous studies have shown contrasting HRV and BRS responses in early diabetes. Maeda et al. (1995) assessed HRV and BRS at day 5 post-induction and reported reduced HRV and BRS, indicating early autonomic suppression [31]. Similar changes have been noted in chronic diabetic rats (up to 70 days and 90 days) [27,32].

In contrast, our study on day 1 found increased HRV and BRS, which suggests that autonomic dysfunction does not develop immediately but rather progresses over time. The methodological differences in pharmacological intervention BRS between these studies may contribute to the discrepancies. Maeda et al. employed autonomic blockade (methylatropine and propranolol), which directly suppresses autonomic function, whereas our study used phenylephrine-induced baroreflex responses, potentially allowing for differential interpretations of autonomic function. Similarly, Dall’Ago et al. (1997) assessed HRV and BRS at day 15 post-induction and found decreased HRV, BRS, BP, and HR [26]. The use of phenylephrine and nitroprusside for baroreflex testing in their study was similar to our approach; however, the time difference (day 1 vs. day 15) suggests that autonomic function dynamically changes during early diabetes progression, further supporting the biphasic hypothesis of CAN development.

A biphasic autonomic pattern has been proposed in early diabetic CAN, where an initial enhancement of parasympathetic activity compensates for metabolic stress before transitioning to autonomic dysfunction. This pattern is supported by findings in autonomic regulation disorders, where early-stage vagal hyperactivity is followed by sympathetic predominance and eventual autonomic failure [33]. Our study, which assessed cardiovascular autonomic function at the immediate onset of diabetes (day 1 post-STZ induction), found increased LFP, HFP, and LHR in hyperglycemic diabetic rats compared to controls. These findings align with the observed reduction in BP and HR, suggesting that parasympathetic hyperactivity serves as a compensatory mechanism to maintain cardiovascular stability during early diabetes. Thus, our findings provide novel insights into the early phase of diabetic CAN, highlighting that autonomic dysfunction is not immediate upon diabetes onset but follows a biphasic trajectory. Increased HRV and BRS at day 1 may serve as an early compensatory mechanism, which eventually transitions into autonomic failure as the disease progresses.

The Diabetic-2 rat exhibited a marked reduction in both LF_Coh_ and HF_Coh_ during the hyperglycemic state. This reduction reflects compromised baroreflex coupling and impaired autonomic regulation, which are characteristic features of early-stage CAN in diabetes. Following insulin administration and the subsequent normalization of blood glucose, both LF_Coh_ and HF_Coh_ showed notable increases, suggesting a partial restoration of autonomic coordination between cardiovascular signals (Figure 3). This individual observation supports the hypothesis that autonomic dysfunction in early diabetes may be at least partially reversible with timely glycemic control.

Consistent with this case, the broader group of diabetic rats exhibited a trend toward normalization of cardiovascular parameters, including SBP, DBP and HR, after the correction of hyperglycemia. In contrast to conventional BRS assessments such as BRS_spn_ and BRS_phe_, our data demonstrated that frequency-domain coherence LF_Coh_ and MS-CXApEn at all scale levels revealed statistically significant impairments in hyperglycemic diabetic rats. Although post-insulin treatment changes did not reach statistical significance at the group-level changes, individual trajectories revealed consistent trends toward recovery. This apparent discrepancy—where coherence analysis shows significant impairment while BRSspn and BRSphe remain statistically unchanged—may be attributed to the differing sensitivities and methodological properties of these measures. Unlike the Oxford method, which depends on pharmacologically induced blood pressure fluctuations and may fail to capture the full spectral range of baroreflex activity, frequency-domain spectral analysis offers a continuous, non-invasive assessment that is better suited to a variety of physiological conditions. Although the sequence method is valued for its simplicity and ease of application, it has been shown to overestimate BRS and is particularly vulnerable to respiratory influences. Respiratory-induced fluctuations in intrathoracic pressure and vagal tone can introduce variability that compromises the accuracy of spontaneous baroreflex gain derived from this approach [34,35]. In contrast, frequency coherence maintains robust performance across a range of physiological states, with coherence values frequently remaining above statistical thresholds even under conditions such as hypoxia or physical stress [36].

The partial reversibility observed suggests that early intervention may mitigate the progression of CAN. However, the modest extent of recovery also underscores the likelihood that early stress-induced vagal imbalance, oxidative stress, and subclinical neural alterations may persist and require more time or multimodal treatment to resolve. We speculate that the early improvements may result from a rapid attenuation of oxidative or inflammatory responses following euglycemia restoration—potentially attributable not only to glycemic normalization but also to the direct physiological actions of insulin itself [37]. Although the exact mechanisms remain uncertain, these findings highlight the remarkable sensitivity and plasticity of the autonomic cardiovascular system during the initial phase of diabetes. Furthermore, the relatively short post-insulin observation window (~1 h) may have been insufficient to capture delayed recovery processes. This suggests that there may exist a short therapeutic window during which early interventions could potentially prevent or delay the development of CAN and its associated complications. There is limited direct evidence from recent studies specifically proving that insulin therapy alone prevents or reverses CAN. However, indirect findings strongly suggest that tight glycemic control which insulin can help achieve is crucial in reducing the risk or progression of CAN. In the DCCT/EDIC study, patients with type 1 diabetes who received intensive insulin therapy had significantly lower incidences of CAN after long-term follow-up compared to those on conventional therapy [38]. This reinforces the importance of early and sustained glycemic intervention to attenuate the evolution of autonomic dysfunction.

Our findings carry important clinical implications. Coherence analysis provides a sensitive and frequency-resolved approach for detecting early diabetic CAN even before conventional baroreflex indices become abnormal. As a non-invasive technique, it holds promise as a practical tool for early screening, longitudinal monitoring, and timely intervention in diabetic patients at risk for developing CAN. Translationally, this approach enables clinicians to identify subclinical autonomic dysfunction before overt symptoms or irreversible damage occur. For diabetic populations particularly those at risk of CAN routine assessment of BRS via spectral coherence could guide earlier intervention strategies, such as tighter glycemic control, cardiovascular risk reduction, and targeted autonomic modulation therapies. Ultimately, integrating BRS assessment into the diabetes care system may enhance risk stratification, enable personalized management, and potentially delay or prevent the progression of autonomic complications [39,40].

Moreover, our results emphasize the potential reversibility of early baroreflex impairment and highlight the superior sensitivity of nonlinear and coherence-based metrics in detecting subclinical autonomic dysfunction, particularly in cases where traditional BRS measures fail to reveal such changes. These findings underscore the clinical importance of early and sustained glycemic control in mitigating the progression of CAN and other diabetes-related complications. Further longitudinal studies are needed to delineate the temporal dynamics of autonomic impairment and to determine optimal windows for therapeutic intervention.

## 5. Study Limitations

This study has several limitations that should be acknowledged. First, given the limited sample size and biological homogeneity of inbred WKY rats under controlled conditions, a formal test for data normality was not initially performed prior to ANOVA. The relatively small number of animals may limit the statistical power for detecting subtle differences among groups. However, consistent trends were observed across multiple cardiovascular autonomic parameters, supporting the robustness of the findings. All the *p* values were demonstrated in Table 2 and Table 3. In accordance with the American Statistical Association’s 2016 guidelines, we also clarified the interpretation of *p*-values in the range of 0.05 to 0.10 as potential trends, particularly in the context of exploratory or small-sample studies [41]. Second, the study focused on a short-term model of streptozotocin-induced diabetes, primarily capturing early-stage alterations in autonomic function. While this design allowed for the identification of potential early biomarkers, the long-term trajectory of these changes warrants further investigation. Third, although coherence analysis and nonlinear metrics such as MS-CXApEn provided valuable insights, additional physiological correlates—such as direct recordings of sympathetic or parasympathetic nerve activity—were not available. Lastly, the influence of anesthesia on autonomic tone, though minimized by protocol standardization, cannot be entirely excluded. Despite these limitations, the study offers novel observations regarding the reversibility of early autonomic impairment and highlights the potential utility of coherence analysis as a sensitive method for early detection of CAN. Further studies with larger cohorts, longer follow-up, and clinical validation are encouraged to expand upon these findings.

## 6. Conclusions

In conclusion, this study underscores the impact of hyperglycemia on cardiovascular parameters and highlights the potential benefits of glycemic correction in partially restoring cardiovascular function in diabetic rats. These findings reinforce the critical role of stringent blood sugar control in reducing the burden of diabetic complications, including CAN.

## Figures and Tables

**Table 1:** Physiological characteristics of the animals.

	Normal *n* = 8	DM *n* = 8	
		Hyperglycemic	Euglycemic
Body weight (g)	303.75 ± 24.83 *	265.25 ± 35.90	265.25 ± 35.90
Blood sugar (mg/dL)	104.25 ± 10.98 *	360.63 ± 63.51 ^#^	183.13 ± 19.74
SBP (mmHg)	154.85 ± 16.44 *	134.53 ± 23.62	151.18 ± 17.15
DBP (mmHg)	103.79 ± 20.32 *	80.83 ± 25.27	87.85 ± 13.14
HR (beats)	369.38 ± 34.12 *	338.13 ± 26.71	346.63 ± 19.68

Values are expressed as mean ± SD. HR: heart rate, SBP: systolic blood pressure, DBP: diastolic blood pressure, * *p* < 0.05 indicates a statistically significant difference between normal controls and hyperglycemic diabetic rats. ^#^ *p* < 0.05 indicates a statistically significant difference between hyperglycemic diabetic rats and euglycemic diabetic rats following insulin treatment. Significances of difference were assessed using the nonparametric Mann–Whitney U test and ANOVA with Fisher’s least significant difference post hoc test.

**Table 2:** Evaluations of cardiovascular autonomic function in control and diabetic rats under hyperglycemic and euglycemic conditions.

	Normal	DM Hyperglycemic	*p* Value
	*n* = 8	*n* = 8	
BRS_spn_ (ms/mmHg)	0.82 ± 0.58	1.04 ± 0.94	1.000
BRS_phe_ (ms/mmHg)	0.82 ± 0.50	0.77 ± 0.67	0.753
LFP	0.29 ± 0.19 *	0.84 ± 0.64	0.012
HFP	1.55 ± 0.74	2.57 ± 1.90	0.401
LHR	0.20 ± 0.15 *	0.35 ± 0.10	0.027
MS-CXApEn_small_	1.23 ± 0.24 *	1.62 ± 0.13	0.002
MS-CXApEn_medium_	1.01 ± 0.28 *	1.29 ± 0.12	0.021
MS-CXApEn_large_	0.91 ± 0.27 *	1.17 ± 0.12	0.036
LF_Coh_	0.14 ± 0.07 *	0.08 ± 0.04	0.046
HF_Coh_	0.19 ± 0.06	0.16 ± 0.10	0.208

BRS_spn_: spontaneous baroreflex sensitivity; BRS_phe_: baroreflex sensitivity assessed via intravenous injection of phenylephrine; LFP: low-frequency power derived from fast Fourier transform (FFT); HFP: high-frequency power derived from FFT; LHR: ratio of LFP to HFP; MS-CXApEn_small_: averaged multiscale cross-approximate entropy between SBP and RRI from scales 1 to 3; MS-CXApEn_medium_: averaged multiscale cross-approximate entropy between SBP and RRI from scales 4 to 6; MS-CXApEn_large_: averaged multiscale cross-approximate entropy between SBP and RRI from scales 7 to 10; LF_Coh_: low-frequency coherence between systolic blood pressure and R-R intervals; HF_Coh_: high-frequency coherence between systolic blood pressure and R-R intervals. * Significances of difference were determined as *p* < 0.05 by ANOVA with Fisher’s least significant difference post hoc test.

**Table 3:** Evaluations of cardiovascular autonomic function in diabetic rats under hyperglycemic and euglycemic conditions.

	DM *n* = 8		*p* Value
	Hyperglycemic	Euglycemic	
BRS_spn_ (ms/mmHg)	1.04 ± 0.94	1.40 ± 1.52	0.462
BRS_phe_ (ms/mmHg)	0.77 ± 0.67	1.03 ± 1.14	0.753
LFP	0.84 ± 0.64	0.63 ± 0.42	0.529
HFP	2.57 ± 1.90	3.60 ± 3.07	0.462
LHR	0.35 ± 0.10	0.21 ± 0.12	0.059
MS-CXApEn_small_	1.62 ± 0.13	1.51 ± 0.30	0.600
MS-CXApEn_medium_	1.29 ± 0.12	1.21 ± 0.24	0.916
MS-CXApEn_large_	1.17 ± 0.12 *	1.01 ± 0.14	0.027
LF_Coh_	0.08 ± 0.04	0.11 ± 0.04	0.074
HF_Coh_	0.16 ± 0.10	0.24 ± 0.12	0.141

BRS_spn_: spontaneous baroreflex sensitivity; BRS_phe_: baroreflex sensitivity assessed via intravenous injection of phenylephrine; LFP: low-frequency power derived from fast Fourier transform (FFT); HFP: high-frequency power derived from FFT; LHR: ratio of LFP to HFP; MS-CXApEn_small_: averaged multiscale cross-approximate entropy between SBP and RRI from scales 1 to 3; MS-CXApEn_medium_: averaged multiscale cross-approximate entropy between SBP and RRI from scales 4 to 6; MS-CXApEn_large_: averaged multiscale cross-approximate entropy between SBP and RRI from scales 7 to 10; LF_Coh_: low-frequency coherence between systolic blood pressure and R-R intervals; HF_Coh_: high-frequency coherence between systolic blood pressure and R-R intervals. * Significances of difference were determined as *p* < 0.05 by ANOVA with Fisher’s least significant difference post hoc test.

## Data Availability

The datasets used and/or analyzed during this study are available from the corresponding author upon reasonable request.
